# Immune and hematologic alterations associated with CD4^+^ T-cell depletion in people living with HIV in Morocco

**DOI:** 10.1186/s12981-026-00856-7

**Published:** 2026-02-26

**Authors:** Zakaria Elkodmiri, Abdelmouine Salami, Moulay Yassine Belghali, Rajaa Hazime, Fatima-Ezzohra Eddehbi, Saad Lamjadli, Salma Rouhi, Wafaa Qiddi, Sanaa Sayagh, Malika Idalene, Maryame Ahnach, Noura Tassi, Bouchra Ghazi, Brahim Admou

**Affiliations:** 1 Immunopathology-Immunotherapy-Immunomonitoring Laboratory, Faculty of Medicine, Mohammed VI University of Health and Sciences (UM6SS), Casablanca, Morocco; 2 Laboratory of Immunology, Center of Clinical Research, Mohammed VI University Hospital, Marrakech, Morocco; 3 Laboratory of Hematology, Faculty of Medicine and Pharmacy, Mohammed VI University Hospital, Cadi Ayyad University, Marrakech, Morocco; 4 Department of Infectious Diseases, Faculty of Medicine and Pharmacy, Mohammed VI University Hospital, Cadi Ayyad University, Marrakech, Morocco; 5 Department of Hematology, Cheikh Khalifa International University Hospital, Casablanca, Morocco; 6 Laboratory of Biotechnology, Conservation and Valorization of Bioresources, Faculty of Sciences Dhar El Mehraz, Sidi Mohamed Ben Abdellah University, Fez, Morocco; 7 Biosciences Research Laboratory, Faculty of Medicine and Pharmacy, Cadi Ayyad University, Marrakech, Morocco

**Keywords:** People living with HIV, CBC, CD4 + T-cell depletion, Hematological changes, Immunophenotyping, flow cytometry

## Abstract

HIV infection profoundly impacts both the immune and hematologic systems. This retrospective study aimed to assess the effect of CD4^+^ T-cell depletion on peripheral immune and hematologic cell populations in 1293 people living with HIV (PLWH). Patients with CD4^+^ counts < 200 cells/µL exhibited significant reductions in neutrophils (4000 ± 1200 cells/µL), eosinophils (150 ± 50 cells/µL), basophils (30 ± 10 cells/µL), monocytes (300 ± 100 cells/µL), erythrocytes (4.3 ± 0.6 × 10^6^/µL), and platelets (268 ± 78 × 10^3^/µL). As evaluated in 93 PLWH cases, B cells showed a similar trend (150 ± 50 cells/µL), while the decrease of NK cells was not significant (200 ± 70 cells/µL),. In parallel, we observed a compensatory increase in CD8^+^ T lymphocytes (850 ± 300 cells/µL). These findings highlight the complex interplay between CD4^+^ T-cell depletion and both immune and hematologic cells in the context of HIV infection, which emphasizes the need for integrated monitoring to inform targeted interventions and optimize clinical outcomes.

**Supplementary Information:**

The online version contains supplementary material available at 10.1186/s12981-026-00856-7.

## Background

Human immunodeficiency virus (HIV) is a retrovirus that primarily targets the immune system, infecting CD4^+^ T cells and progressively impairing immune function. The most advanced stage of infection, acquired immunodeficiency syndrome (AIDS), is defined by CD4^+^ T-cell counts below 200 cells/µL or the presence of AIDS-defining illnesses [1]. HIV infection induces chronic immune activation and persistent viral replication, leading to CD8^+^ T-cell expansion, elevated proinflammatory cytokines, and gradual CD4^+^ T-cell depletion, collectively compromising the host’s ability to mount effective immune responses against infections and malignancies [2].

In addition to immune dysfunction, hematological abnormalities are among the most common complications in individuals with HIV/AIDS, often occurring even before the initiation of antiretroviral therapy [3]. Dysregulation of eosinophils, basophils, and B cells impairs both innate and humoral immunity, while disruptions in erythropoiesis, granulocyte production, and platelet function increase the risk of anemia, leukopenia, and thrombocytopenia. These interconnected disturbances underscore the close interplay between immune deterioration and hematologic imbalance [4]. Therefore, this study aimed to comprehensively assess immune and hematologic cell populations in people living with HIV (PLWH) at diagnosis, with particular attention to variations associated with severe CD4^+^ T-cell depletion.

## Methods

### Study population and design

This retrospective study was conducted at the Immunology Department in collaboration with the Infectiology and Hematology Departments of Mohammed VI University Hospital of Marrakech. The study included 1293 people living with HIV (PLWH) at the time of diagnosis, who were admitted to and monitored in the Clinical Infectious Diseases Department. HIV infection was confirmed in all patients using conventional techniques (ELISA and Western blot). Inclusion criteria consisted of confirmed HIV infection and availability of baseline laboratory data at diagnosis, including complete blood count (CBC) and CD4^+^ T-cell measurements. All participants were treatment-naïve, and common HIV-associated coinfections were not excluded. All participants were treatment-naïve, and measurements were performed before initiation of antiretroviral therapy. All age groups were represented. Exclusion criteria were limited to missing essential laboratory data or patients already receiving treatment. Patients originated from both urban and rural areas, representing a broad spectrum of socioeconomic backgrounds, including variations in income, education, and access to healthcare services.

Blood samples were collected in EDTA tubes, and patients were divided into two groups. Group 1 (1200 patients) underwent CBC using a Sysmex XP-300^™^ analyzer and CD4^+^ T-cell counts by flow cytometry (FACSCanto II, BD Biosciences) using a Tritest panel (CD45-PerCP, CD3-FITC, CD4-PE). Standard incubation and lysis protocols were followed to ensure reproducibility. Group 2 (93 patients) underwent CBC and detailed T, B, and NK cell immunophenotyping using the BD six-color TBNK reagent (CD45, CD3, CD4, CD8, CD16 + CD56, CD19), processed according to manufacturer-recommended lysis, washing, and resuspension steps, and analyzed on the FACSCanto II using standard gating strategies. For analysis, patients were stratified by baseline CD4^+^ T-cell count into four categories: <200, 200–500, 500–1500, and > 1500 cells/µL. This stratification enabled comparison of immune and hematologic alterations across different levels of immune suppression, providing insights into the relationship between HIV-related immune status and hematologic changes at diagnosis.

### Statistical analysis

Descriptive statistics (mean ± SD) were calculated for all immune and hematologic parameters across CD4^+^ T-cell strata (< 200, 200–500, 500–1500, and > 1500 cells/µL). Data distribution was assessed using the Kolmogorov–Smirnov test. Comparisons between two clusters (< 200 vs. ≥200 cells/µL) were performed using the Student’s t-test. When comparing three or more CD4^+^ strata, a one-way ANOVA followed by Tukey’s honestly significant difference (HSD) post-hoc test was used for multiple comparisons. Statistical significance was defined as *p* < 0.05. Pearson’s correlation coefficient was employed to evaluate associations between CD4^+^ counts and other immune or hematologic parameters.

### Ethical considerations

The study was conducted in accordance with the ethical principles of the Declaration of Helsinki for research involving human subjects. It was based on a retrospective analysis of patient data extracted anonymously from the electronic database of the immunology and hematology departments, under the supervision of the department heads. All tests performed on patients were part of their routine care, and no additional investigations were conducted on patient samples. Therefore, approval from the local ethics committee was not deemed necessary.

## Results

Table 1 showed that the whole study population had a mean age of 38.8 ± 10 years (range: 35–55). There was a male predominance (55.1%), with a sex ratio of 1.22. We observed a significant negative correlation between age and CD4^+^ T-cell count (*r* = − 0.32, *p* = 0.001), indicating that CD4^+^ levels decline progressively with increasing age. Consistently, individuals over 56 years presented markedly lower mean CD4^+^ counts (535 ± 18 cells/µL) compared with those aged 36–50 years (628 ± 110 cells/µL; *p* < 0.01).

**Table 1:** Distribution of PLWH according to socio-demographic and clinical characteristics according to CD4 + T-cell clusters

TCD4 Clusters	Group I (< 200 cells/µL)	Group II (200–500 cells/µL)	Group III (500–1500 cells/µL)	Group IV (> 1500 cells/µL)	Total
n	191	351	677	74	1293
Socio-demographic characteristics					
Age*	40.7	40.2	38.0	34.0	38.8
Gender M/F*	105/86	194/157	365/312	44/30	713/580
Origin (U/R)*	171/20	301/50	528/149	40/34	1040/253
Clinical Characteristics					
Common or specific symptoms					
Diarrhea associated with weight loss	95	55	25	6	181 (13.9%)
Weakness or cachexia	48	14	4	0	66 (5.1%)
Oropharyngeal candidiasis	40	18	3	0	61 (4.7%)
Prolonged fever	30	14	6	0	50 (3.8%)
Diffuse and persistent lymphadenopathy	18	7	3	0	28 (2.1%)
Opportunistic infections					
Tuberculosis	75	50	20	2	147 (11.4%)
Cryptococcus meningitis	24	5	0	0	29 (2.2%)
Oesophageal candidiasis	38	12	0	0	50 (3.8%)
Kaposi sarcoma	17	0	0	0	17 (1.3%)
Pneumocystosis	35	9	0	0	44 (3.4%)
Other/Unknown conditions	10	11	9	9	39 (3%)

*CD4: cluster of differentiation 4; PLWH: people living with HIV, M/F: Male/Female, U/R, Urban/Rural, p: p value, r: Pearson correlation coefficient

Only parameters showing relevant variations with CD4^+^ T-cell counts are displayed in tables, while those that did not vary significantly, such as NK (CD16^+^CD56^+^) cells, are mentioned in the text.

Table 2 summarizes hematologic and immune parameters in PLWH, including red blood cells, platelets, neutrophils, eosinophils, basophils, monocytes, CD8^+^ T cells, CD3^+^ T cells, and B cells (CD19^+^), presented as mean values for each CD4^+^ T-cell cluster (< 200, 200–500, 500–1500, and > 1500 cells/µL). This table provides a descriptive overview of trends in hematologic and lymphocyte subpopulations relative to immune status, highlighting changes associated with increasing CD4^+^ T-cell counts. NK (CD16^+^CD56^+^) cells had a mean of 200 ± 70 cells/µL, with mean counts per TCD4 cluster of < 200: 186 cells/µL, 200–500: 233 cells/µL, 500–1500: 291 cells/µL, and > 1500: 261 cells/µL. Tukey’s HSD tests showed that PLWH with CD4^+^ <200 cells/µL had significantly lower hematologic and lymphocyte values compared with one or more higher CD4^+^ clusters. CD4^+^ T-cell counts differed across all clusters (*p* < 0.05, several comparisons *p* < 0.01).

**Table 2:** Immune and hematologic parameters in PLWH according to CD4 + T-cell clusters

Parameters	Mean ± SD [Reference interval]	Group I (< 200 cells/µL)	Group II (200–500 cells/µL)	Group III (500–1500 cells/µL)	Group IV (> 1500 cells/µL)
Complete Blood Count					
Red blood cells (×10^6^/µL)	4.3 ± 0.6 [3.7–4.9]	4.7 ± 0.5	4.56 ± 0.35	4.59 ± 0.30	4.62​​± 0.25
Platelets (×10^3^/µL)	268 ± 78 [190–346]	235 ± 60	264 ± 70	272 ± 75	300 ± 80
Neutrophils (cells/µL)	4000 ± 1200 [2800–5200]	3771 ± 900	3685 ± 950	4008 ± 1000	4455 ± 1100
Eosinophils (cells/µL)	150 ± 50 [100–200]	152 ± 50	177 ± 55	168 ± 50	249 ± 70
Basophils (cells/µL)	30 ± 10 [20–40]	18 ± 7	29 ± 9	30 ± 10	42 ± 12
Monocytes (cells/µL)	300 ± 100 [200–400]	250 ± 80	300 ± 90	300 ± 95	300 ± 100
Immunophenotyping					
CD4 + T (cells/µL)	450 ± 180 [270–630]	120 ± 50	350 ± 90	1027 ± 250	1000 ± 240
CD8 + T (cells/µL)	850 ± 300 [550–1150]	804 ± 250	1034 ± 260	1027 ± 250	1000 ± 240
CD3 + T (cells/µL)	1350 [1000–2200]	891 ± 300	1378 ± 350	1887 ± 400	1617 ± 380
B (CD19+) (cells/µL)	150 ± 50 [100–200]	155 ± 90	296 ± 80	381 ± 90	328 ± 85

*CD4 T: CD4⁺ T lymphocytes, CD8 T: CD8⁺ T lymphocytes, CD3 T: total T lymphocytes, CD19: B lymphocytes, NK: natural killer cells (CD16⁺CD56⁺), PLWH: people living with HIV, SD: standard deviation, RBC: red blood cells, p: p value, Tukey’s HSD: Tukey’s honestly significant difference test. Values are expressed as mean ± standard deviation (SD). Reference intervals are laboratory normal ranges

Table 3 compares hematologic and lymphocyte parameters in PLWH stratified by CD4^+^ counts (< 200 vs. ≥200 cells/µL). Only parameters demonstrating significant differences or correlations with CD4^+^ counts are included. The table shows reductions in neutrophils, eosinophils, basophils, red blood cells, platelets, and B cells, alongside an increase in CD8^+^ T cells, correlating with lower CD4^+^ counts (< 200 cells/µL). Parameters that did not vary notably, such as NK cells (CD16^+^CD56^+^) (*p* = 0.05, t = 2.98; *r* = 0.21, Pearson correlation), showed no association with CD4^+^ counts.

**Table 3:** Comparative analysis of hematologic and lymphocyte parameters in PLWH by CD4^+^ count (< 200 vs. ≥200 cells/µL)

Parameters	<200 cells/µL (Mean ± SD)	≥200 cells/µL (Mean ± SD)	*p*-value (group comparison)	*r* (correlation with CD4^+^)	*p*-value (correlation)
Neutrophils (×10^3^/µL)	3.2 ± 0.9	4.1 ± 1.0	0.002	0.45	0.001
Eosinophils (×10^3^/µL)	0.15 ± 0.05	0.18 ± 0.06	0.045	0.38	0.004
Basophils (×10^3^/µL)	0.018 ± 0.007	0.030 ± 0.010	0.015	0.4	0.003
Red blood cells (×10^6^/µL)	3.9 ± 0.5	4.4 ± 0.6	0.01	0.42	0.002
Platelets (×10^3^/µL)	210 ± 60	275 ± 80	0.005	0.48	0.001
CD8^+^ T (cells/µL)	950 ± 250	800 ± 220	0.001	− 0.30	0.01
B (CD19^+^) (cells/µL)	320 ± 90	240 ± 80	0.004	− 0.32	0.008

*CD4 T: CD4^+^ T lymphocytes, CD8 T: CD8^+^ T lymphocytes, CD19: B lymphocytes, NK: natural killer cells (CD16^+^CD56^+^), PLWH: people living with HIV, SD: standard deviation, r: Pearson correlation coefficient

## Discussion

AIDS is the advanced stage of HIV infection and is defined by a CD4^+^ T-cell count below 200 cells/µL, according to the CDC (Center for Disease Control) classification. People with CD4^+^ counts below this level are very vulnerable to severe infections and other immune-related problems. In this study, many participants had CD4^+^ counts under 200 cells/µL, which is important for understanding the clinical and immune outcomes. CD4^+^ T-cell count is also used to start antiretroviral therapy and to monitor treatment response. The decline in CD4^+^ T cells during HIV infection can result from direct viral damage, destruction by the immune system, and reduced production of T cells in the thymus [5].

The data presented in the current study showed that CD4^+^ T-cell counts declined with increasing age. This trend reflects natural age-related declines in CD4^+^ T cells and, due to local factors such as limited HIV testing, low perceived risk, and delays in seeking care at our regional hospital serving the entire area, contributes to lower immune status at diagnosis. This decline may be explained also by thymic involution, immunosenescence, and reduced T-cell regenerative capacity [6]. Male patients showed a nonsignificant trend toward lower CD4^+^ counts than females (means = 430 vs. 470 cells/µL), which could be consistent with estrogen-mediated immunoprotection [7]. These findings suggest that age- and sex-specific CD4^+^ monitoring may be informative in PLWH.

PLWH with < 200 CD4^+^ T-cells/µL exhibited significant reductions in neutrophils, eosinophils, basophils, monocytes, red blood cells, and platelets, alongside increases in CD8^+^ T cells, which goes in line with alterations in immune and hematologic compartments. Declines in neutrophils and eosinophils indicate potential compromises in antibacterial and anti-parasitic defenses, likely influenced by disrupted G-CSF, IL-8, IL-5 signaling, and overexpression of TGF-β, further modulated by pro-inflammatory cytokines, such as TNF-α and IL-6 [8, 9]. Reduced basophils counts may affect the regulation of allergic and inflammatory responses via IL-3, IL-33, and STAT5 pathways [10]. The depletion of monocytes may impact both innate and adaptive immunity, with persistent activation during ART, hence possibly contributing to systemic inflammation and cardiovascular risk via IL-1β and TNF-α signaling [11].

Our results also highlighted dysfunctions in the adaptive immune system. In fact, CD8^+^ T-cell count increased inversely with CD4^+^ levels, reflecting a known compensatory response to maintain antiviral activity through IL-2 and IL-15 signaling via JAK-STAT pathways [12]. B-cell counts decreased with CD4^+^ depletion, suggesting potential impairment of humoral immunity, with increased susceptibility to infections, probably related to disrupted CD40 and cytokine receptor signaling (IL-4, IL-21) and alterations in PI3K-Akt and JAK-STAT pathways [13].

Hematologic changes were evident among patients with severe CD4^+^ depletion. Indeed, red blood cell counts were reduced, leading to anemia through different mechanisms, such as chronic inflammation, opportunistic infections, and direct viral interference with erythropoiesis [14, 15]. Dysregulated TNF-α and IL-6 signaling may impair erythroid progenitor survival, while hepcidin overexpression can compromise iron homeostasis [16, 17]. The platelet count was similarly reduced, with the risk of thrombocytopenia and hemorrhagic complications, likely driven by bone marrow suppression, immune-mediated destruction, and impaired megakaryopoiesis via TNF-α, IL-6, and TGF-β pathways [17].

These results indicate that CD4^+^ T-cell depletion in PLWH is accompanied by alterations in both immune and hematologic compartments. Monitoring these parameters provides useful information about the patient’s immune status, with an extensive overview on potential associated hematological abnormalities, which can help inform clinical assessment and patient management [18]. Co-infections remain an important consideration PLWH, as the immunosuppression caused by declining CD4^+^ T-cell counts increases susceptibility to opportunistic infections. In this study, while HIV infection itself drives immune dysfunction, its presence may further impair hematologic and immunologic abnormalities [19]. Indeed, routine measurements of whole blood cells can detect anemia, leukopenia, thrombocytopenia or other blood abnormalities, while detailed immune cell phenotyping, including T cell subsets (especially CD3^+^ and CD4^+^ T cells) and B cells, can reveal immune deficiencies, thus helping to evaluate disease severity, and inform decisions about treatment initiation or modification [20]. Together, these measures give physicians a clearer picture of disease status and patient resilience.

### Study limitations

Despite the relevance of these findings, several limitations should be acknowledged. Only a subset of PLWH underwent comprehensive T, B, and NK cell phenotyping, limiting generalizability of observed results. The lack of additional immunological markers and complete clinical data constraints the depth of analysis. Plasma HIV-1 RNA viral load was not assessed for all patients due to its high cost and the social circumstances of some participants. The single-center design, encompassing both urban and rural participants, may restrict external validity. Finally, the retrospective nature of the study precludes causal inference. Future research with larger, well-characterized cohorts and expanded immunophenotyping is needed to confirm and extend these observations.

## Conclusion

This study indicates that CD4^+^ count serves as an important marker of immune and hematologic alterations in PLWH, particularly in advanced stages (< 200 cells/µL). CD4^+^ depletion was associated with changes in CD8^+^ and B-cell populations, alongside significant reductions in granulocytes, monocytes, red blood cells, and platelets, which may contribute to increased susceptibility to infections, anemia, and bleeding. The multiparametric analysis illustrates the interconnected changes across immune and hematologic compartments in PLWH, supporting their consideration in patient monitoring and clinical decision-making.

## Supplementary Information

Below is the link to the electronic supplementary material.

Supplementary Material 1.

## Data Availability

Data are available from the corresponding author upon request.

## Abbreviations

PLWH: People living with HIV
ART: Antiretroviral Therapy
CD: Cluster of differentiation
T: T lymphocytes
B: B lymphocytes
NK: Natural killer cells
CBC: Complete blood count
IL: Interleukin
TNF-α: Tumor necrosis factor-alpha
TGF-β: Transforming growth factor-beta
SD: Standard deviation
