# Supplementary material for: Immune and hematologic alterations associated with CD4+ T-cell depletion in people living with HIV in Morocco
Source: AIDS Res Ther. 2026 Feb 26;23:41. doi: 10.1186/s12981-026-00856-7 (PMC13040705; doi:10.1186/s12981-026-00856-7)
Supplement: Supplementary file 1 — Supplementary Material 1. [file 12981_2026_856_MOESM1_ESM.pdf]

Supplementary file

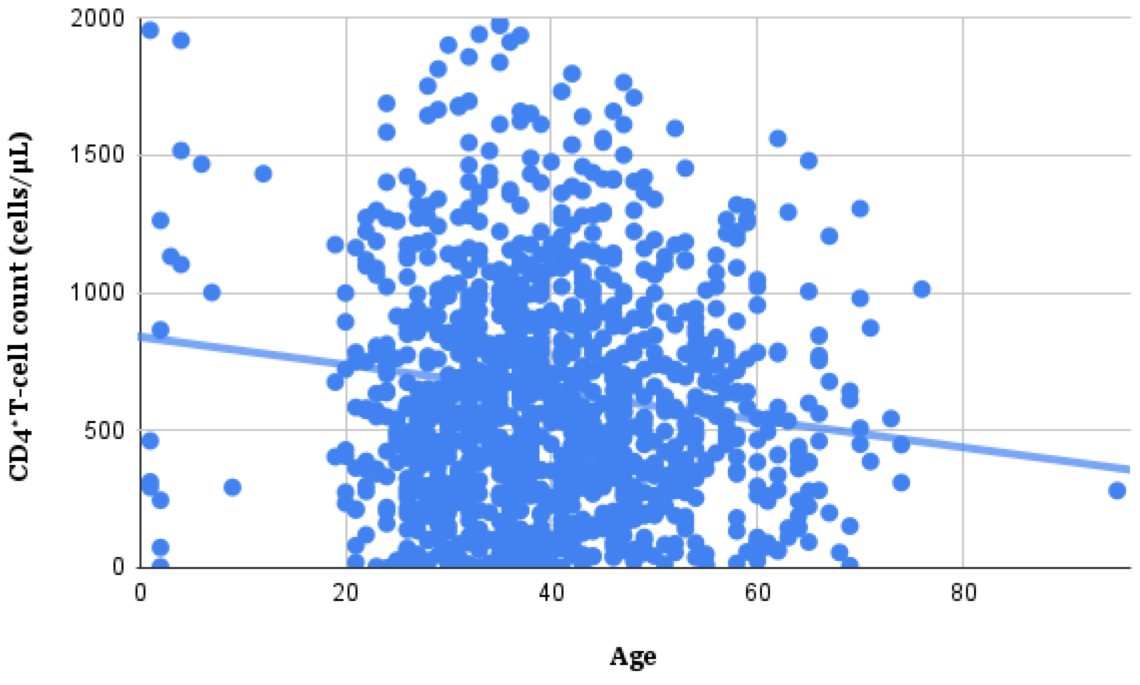

Figure 1: Pearson correlation between age and CD4⁺ T-cell count in PLWH.
**CD4 T: CD4⁺ T lymphocytes, PLWH: people living with HIV, r: Pearson correlation coefficient, p: p value.*

This figure shows the Pearson correlation between age and CD4⁺ T-cell count in PLWH at diagnosis. Each point represents an individual participant. A linear regression line illustrates the inverse relationship, indicating lower CD4⁺ T-cell counts with increasing age. Pearson correlation analysis demonstrated a negative correlation ($r = -0.32$, $p = 0.001$).

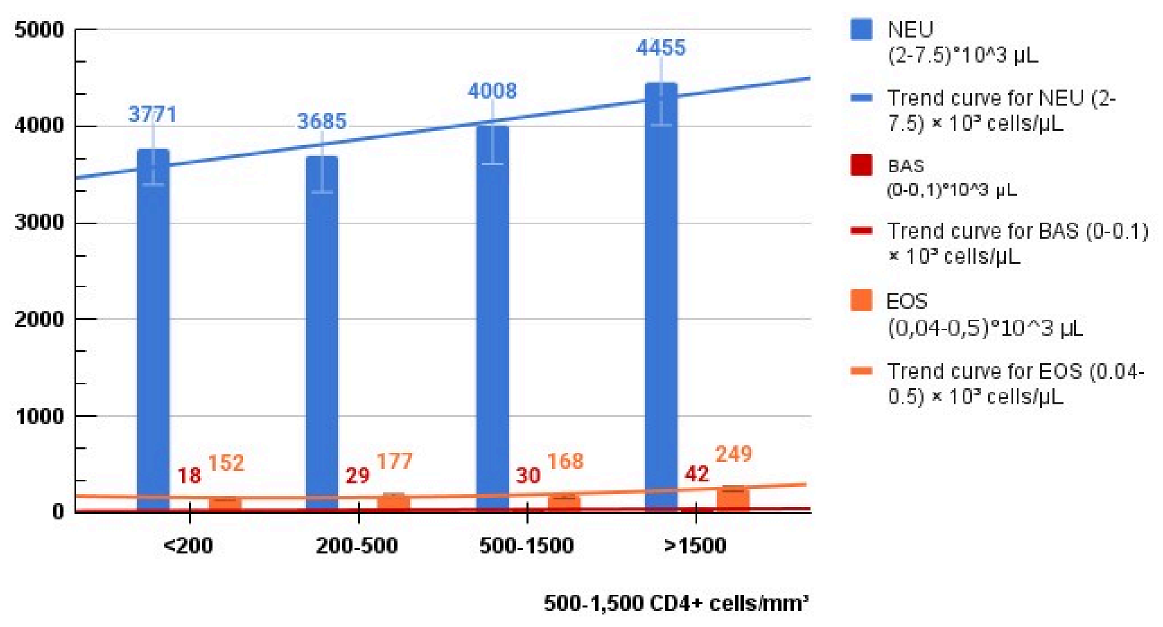

Figure 2: Distribution of mean polymorphonuclear cell populations (Neutrophils, Basophils, Eosinophils) and Monocytes based on CD4 T-Cell cluster levels

**CD4: cluster of differentiation 4, PLWH: people living with HIV, NEU: neutrophils, BAS: basophils, EOS: eosinophils.*

This figure shows a progressive change in mean absolute counts of polymorphonuclear cells including neutrophils, eosinophils, basophils and monocytes across decreasing $CD4^+$ T-cell clusters (<200, 200–500, 500–1500, and >1500 cells/µL) in PLWH at diagnosis. Lower $CD4^+$ T-cell levels are associated with reduced counts of these innate immune cell populations, illustrating a downward trend with increasing immune suppression.

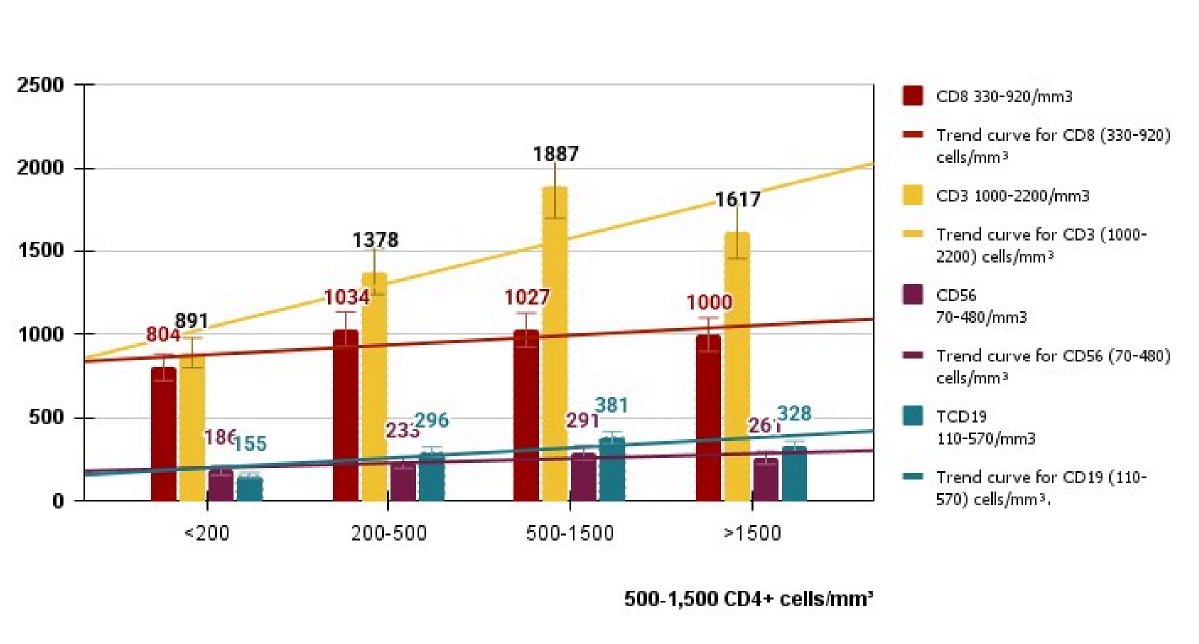

Figure 3: Distribution of mean immunological markers (TCD8, TCD3, B CD19, NK CD16/56) based on CD4 T-Cell cluster levels

**CD: cluster of differentiation, CD4: cluster of differentiation 4, TCD8: $CD8^+$ T lymphocytes, CD3: total T lymphocytes, CD19: B lymphocytes, NK: natural killer cells ($CD16^+CD56^+$), PLWH: people living with HIV.*

This figure illustrates trends in lymphocyte subsets in PLWH across $CD4^+$ T-cell clusters (<200, 200–500, 500–1500, and >1500 cells/µL). Mean TCD8 and total $CD3^+$ T-cell counts increase as $CD4^+$ T-cell levels decrease, whereas B lymphocyte counts show a declining trend with greater $CD4^+$ T-cell depletion. NK cell counts remain relatively stable across clusters.

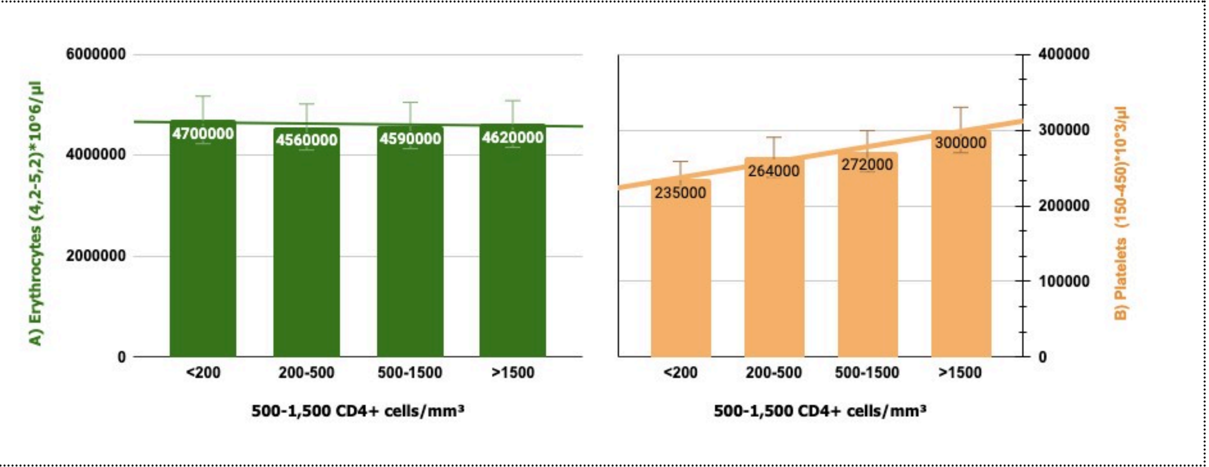

Figure 4: Distribution of hematological cell means (Erythrocytes and Platelets) based on CD4 T-Cell cluster levels

**CD4: cluster of differentiation 4, PLWH: people living with HIV, CBC: complete blood count.*

This figure depicts a decreasing trend in mean erythrocyte (red blood cell) and platelet counts measured by CBC with lower $CD4^+$ T-cell levels across four clusters (<200, 200–500, 500–1500, and >1500 cells/µL). The figure demonstrates the association between advanced $CD4^+$ T-cell depletion and altered hematological parameters in people living with HIV at diagnosis.
